# Altered Phenylpropanoid Metabolism in the Maize *Lc*-Expressed Sweet Potato (*Ipomoea batatas*) Affects Storage Root Development

**DOI:** 10.1038/srep18645

**Published:** 2016-01-04

**Authors:** Hongxia Wang, Jun Yang, Min Zhang, Weijuan Fan, Nurit Firon, Sitakanta Pattanaik, Ling Yuan, Peng Zhang

**Affiliations:** 1 National Key Laboratory of Plant Molecular Genetics, CAS Center for Excellence in Molecular Plant Sciences, Institute of Plant Physiology and Ecology, Shanghai Institutes for Biological Sciences, Chinese Academy of Science, Shanghai 200032, China; 2 Shanghai Key Laboratory of Plant Functional Genomics and Resources, Shanghai Chenshan Plant Science Research Center, Chinese Academy of Science, Shanghai Chenshan Botanical Garden, Shanghai 201602, China; 3 Institute of Plant Sciences, The Volcani Center, Agricultural Research Organization, Bet Dagan 50250, Israel; 4 Department of Plant and Soil Science, University of Kentucky, Lexington, KY 40546, USA

## Abstract

There is no direct evidence of the effect of lignin metabolism on early storage root development in sweet potato. In this study, we found that heterologous expression of the maize leaf color (*Lc*) gene in sweet potato increased anthocyanin pigment accumulation in the whole plant and resulted in reduced size with an increased length/width ratio, low yield and less starch content in the early storage roots. RT-PCR analysis revealed dramatic up-regulation of the genes involved in the lignin biosynthesis pathway in developing storage roots, leading to greater lignin content in the *Lc* transgenic lines, compared to the wild type. This was also evidenced by the enhanced lignification of vascular cells in the early storage roots. Furthermore, increased expression of the β-amylase gene in leaves and storage roots also accelerated starch degradation and increased the sugar use efficiency, providing more energy and carbohydrate sources for lignin biosynthesis in the *Lc* transgenic sweet potato. Lesser starch accumulation was observed in the developing storage roots at the initiation stage in the Lc plants. Our study provides experimental evidence of the basic carbohydrate metabolism underlying the development of storage roots, which is the transformation of lignin biosynthesis to starch biosynthesis.

Sweet potato (*Ipomoea batatas* [L.] Lam), cassava (*Manihot esculenta* Crantz) and potato (*Solanum tuberosum* L.) are the major root and tuber crops and an essential component of subsistence agriculture in terms of guaranteeing food security and improving nutrition status regionally^1^. One of the biggest advantages of sweet potato cultivation is the production of fleshy storage roots with high yield, a complex process that transforms adventitious roots to storage roots and results in the accumulation of a large amount of starch as well as other health-promoting components such as anthocyanins and carotenes^2^,^3^,^4^. Storage root development in sweet potato, as a form of secondary growth, has been intensively studied since the 1920s by the plant anatomist and morphologist Dr. Ernst Artschwager^5^. Typically, the sweet potato root system consists of three different types of roots (fibrous roots, pencil roots and storage roots) that originate from adventitious roots and are distinguishable from each other^2^ (Fig. 1A). The vigorous differentiation of circular vascular cambia derived from both primary vascular cambia and secondary cambia formed around secondary xylem elements promotes the cell division and expansion of thin-walled, parenchyma cells for storage of starch granules, which leads to rapid bulking and starchy tuberous root formation^4^,^5^,^6^. The pencil roots are thickened but heavily lignified with a diameter less than 2 cm (Fig. 1A), which suggests that stele lignification during the early phase/stage of storage root development affects storage root development^7^,^8^,^9^,^10^,^11^. In addition, storage root initiation was proposed by Togari^7^ to be influenced by the balance between cambium propagation and lignification, a process that is affected by genetic, physiological, and environmental factors. Under stressful conditions, e.g., drought and poor soil fertility, sweet potato shows retarded storage root development with more pencil root production^12^,^13^, suggesting that sufficient supply of photo-assimilates from the source to the sink is important for starch metabolism and storage root development.

Although several morphoanatomical studies have been conducted on storage root development in sweet potato, the underlying molecular and physiological mechanisms and their regulation are still unclear^2^,^3^,^14^. Carbon flux, the basis of plant growth, is distributed into various branches between the primary and secondary metabolic pathways and affects the factors involved in plant growth and development, including starch, cellulose, lignin and flavonoids^15^. Recently, transcription profiling of the initiating storage roots and fibrous roots has revealed the down-regulation of lignin biosynthesis and up-regulation of starch biosynthesis, which are considered to be the major events involved in storage root initiation^4^,^11^. Mobilization of carbon flux toward starch biosynthesis has also been suggested in another root crop—cassava^16^. During storage root formation, the transition of carbon flux, from phenylpropanoid biosynthesis to carbohydrate metabolism and starch biosynthesis, is considered an important domestication process from wild ancestor to cultivated varieties of cassava^17^; this is similar to the observations in sweet potato^11^. Recently, key genes regulating storage root formation, such as the Dof zing finger transcriptional factor SRF1, MADS-box protein SRD1 and expansins, have been intensively studied^18^,^19^,^20^. SRF1 regulates carbohydrate metabolism in the storage roots through negative regulation of a vacuolar invertase gene^18^, while SRD1 functions in the formation of storage roots by inducing the proliferation of cambium and metaxylem cells^19^, a process that can be negatively regulated by the IbEXP1 gene^20^. Taken together, recent studies indicate that storage root formation may involve the regulation of lignin and starch biosynthesis.

Several important transcription factors that regulate the biosynthesis pathways of primary and secondary metabolites and finally affect carbon partitioning in plants have been reported^21^,^22^. For example, the expression of several NAC family transcription factor that promote the secondary growth of cell walls can up-regulate lignin biosynthesis in wood^23^,^24^. These factors, however, are observed to be down-regulated during storage root development in sweet potato^25^. Moreover, heterologous expression of the maize leaf color (Lc) transcription factor^26^, basic helix-loop-helix (bHLH) transcription factor which regulates flavonoid pathway genes, caused not only an increase in the anthocyanin and proanthocyanidin pigments, but also a reduction in the size of transgenic apple, tomato, creeping bentgrass, etc.^27^,^28^,^29^,^30^. Several factors have been found to be involved in storage root initiation and development in cassava and sweet potato, but they need further functional validation^11^,^16^. Among these factors, three bHLH-homologues, exhibiting 7- to 1.4-fold down-regulation following storage root initiation, were identified in ‘Georgia Jet’ sweet potato^11^.

In this study, we found that ectopic expression of the maize anthocyanin regulator Lc in sweet potato could elevate the expression of the phenylalanine ammonia-lyase (PAL) gene, as well as the downstream genes cinnamate 4-hydroxylase (*C4H*) and 4 coumaroyl-CoA synthase (*4CL*), in the lignin biosynthesis pathway, leading to lignification in adventitious roots and in developing storage roots. Increased lignification in *Lc*-expressing sweet potato roots was accompanied by significant yield reduction as well as repression of starch accumulation in the developing storage roots. In contrast to the situation in the roots, increased lignification of the canopy did not show a significant effect on plant growth. This study thus suggests a cause-and-effect relationship between increased lignification and reduced storage root yield. Furthermore, the results suggest that lignification competes with starch accumulation for the distribution of photo-assimilates in developing storage roots.

## Results

### Molecular and phenotypic characterization of *Lc* transgenic sweet potato

**Figure 1:**
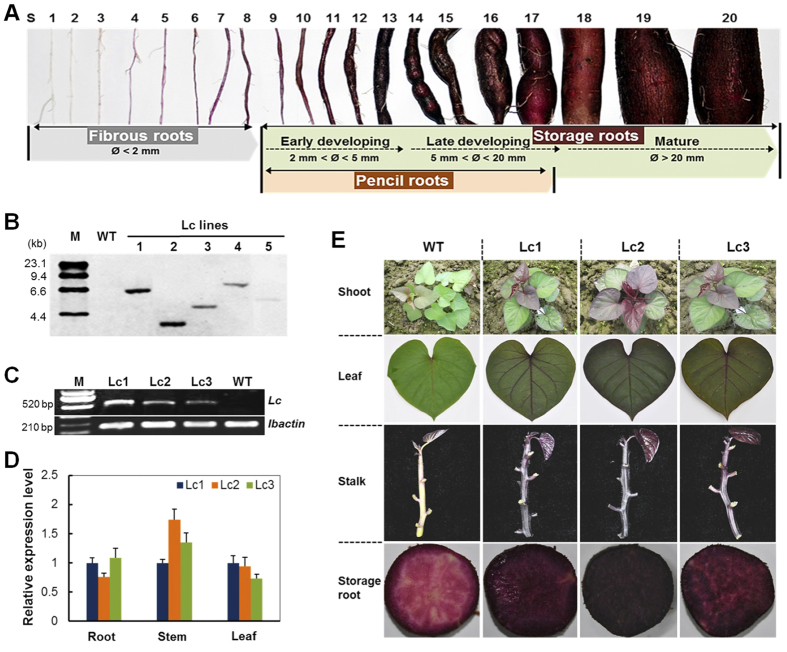
Molecular and phenotypic characterization of wild-type and *Lc* transgenic sweet potato. (**A**) Classification of sweet potato root developmental stages (S1 to S20). S1-S8, Fibrous roots; S9-S13, Early developmental stage of storage roots; S14-S17, Late developmental stage of storage roots; S17–20, Mature storage roots. The pencil roots cover the size of stages from S9 to S17 but are uniformly thickened and lignified. (**B**) Southern blot analysis of *Hin*dIII-digested genomic DNA using the DIG-labeled hygromycin phosphotranferase gene (*hpt*) probe. M, Molecular marker; Lc1–5, independent *Lc* transgenic lines; WT, wild type. (**C**,**D**) Comparison of *Lc* expression levels in the developing storage root (S16), stem and leaf of three 2-month-old *Lc* transgenic lines by RT-PCR (**C**) and qRT-PCR analysis. (**D**) The sweet potato *ACTIN* gene was used as a reference for normalization, and the *Lc* expression levels in Lc1 tissues was used for calibration in qRT-PCR assay. (**E**) Phenotypes of the WT and *Lc* transgenic plants in the field showing the shoots, the 3^rd^ fully expanded leaves from the top, stems and cross-sections of the developing storage roots (S16) after 2 months of growth.

The 1833-bp open reading frame of the maize *Lc* gene driven by the *CaMV* 35S promoter was introduced into purple sweet potato, Ayamurasaki, via *Agrobacterium*-mediated transformation^31^. Twelve independent transgenic plant lines (named the Lc lines) were regenerated and propagated by *in vitro* culturing. The integration number of T-DNA was confirmed by Southern blot analysis using *Hin*dIII-digested genomic DNA hybridized with the digoxygenin (DIG)-labeled probe of the hygromycin phosphotransferase gene (*hpt*). Three independent transgenic lines—Lc1, Lc2 and Lc3—which showed single copy T-DNA integrations (Fig. 1B) were compared for their *Lc* transcription level by RT-PCR (Fig. 1C) and qRT-PCR (Fig. 1D). The three lines showed similar *Lc* expression in the root, stem and leaf, when calibrated with *Lc* expression in the corresponding tissues of the Lc1 plant (Fig. 1D).

The *in vitro* cultured (Supplementary Fig. 1A), greenhouse-grown (Supplementary Fig. 1B) and field-grown plants (Fig. 1E) showed enhanced anthocyanin pigmentation in leaves, stems, adventitious roots and storage roots of *Lc* transgenic plants, compared to the wild type (WT). When harvested from the field after 5 months of growth, all transgenic lines showed dark purple coloration on the whole plant (Fig. 1E; Supplementary Fig. 1C). These results show that heterologous expression of the Lc transcription factor could lead to elevated anthocyanin production in the purple sweet potato.

### Lc promotes flavonoid accumulation and the flavonoid biosynthetic pathway in sweet potato

**Figure 2:**
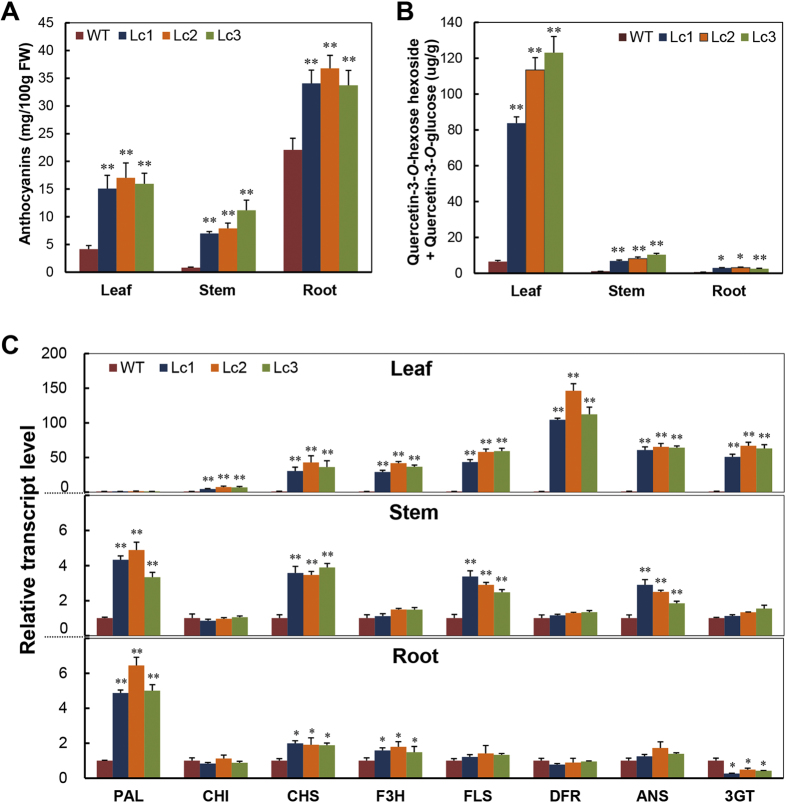
Quantification of anthocyanins and flavonols and transcription levels of flavonoid biosynthetic genes in the leaves, stems and developing storage roots (S16) of 2-month-old wild-type and *Lc* transgenic sweet potato. (**A**,**B**) Changes in the anthocyanin content (**A**) and flavonol (quercetin-3-*O*-hexose-hexoside and quercetin-3-*O*-glucoside) content. (**B**) WT, wild type; Lc1–3, independent *Lc* transgenic lines. (**C**) Transcription levels detected by qRT-PCR analysis. The sweet potato *ACTIN* gene was used as an internal control. PAL, phenylalanine ammonia lyase; CHI, chalcone isomerase; CHS, chalcone synthase; F3H, flavanone 3-hydroxylase; FLS, flavonol synthase; DFR, dihydroflavonol 4-reductase; ANS, anthocyanidin synthase; 3GT, UDP-glucose:flavonoid 3-*O*-glucosyltransferase. Error bars represent the SE of three replicates. Asterisks indicate a significant difference compared to the WT at *P < 0.05 or **P < 0.01 (*t*-test).

Anthocyanin content and expression of anthocyanin pathway genes were measured in the Lc transgenic lines and WT plants. Compared to the WT, the three Lc transgenic lines showed dramatically increased anthocyanin content in leaves, stems and developing storage roots (S16) (Fig. 2A). Anthocyanin content in the Lc1 leaf was 15.09 mg/100 g, 3.6-fold that of the WT (4.16 mg/100 g). The stem of the *Lc* transgenic lines showed the highest fold-change increase in anthocyanin content, 13.63 times that of the WT (0.82 mg/100 g). Further, the anthocyanin content in the storage roots of Lc1, Lc2 and Lc3 was 33.72 mg/100 g to 36.77 mg/100 g, 1.5 times that of the WT (22.09 mg/100 g). Only slight variations in the anthocyanin content were observed between the three transgenic lines (Fig. 2A); these observations reflect the phenotype changes observed in the leaves, stems and storage roots (Fig. 1E).

To investigate the impact of *Lc* expression on the content of other flavonoids in sweet potato, two flavonols—quercetin-3-*O*-hexose-hexoside ([M-H]-625) and quercetin-3-*O*-hexoside ([M-H]-463)—that were recently identified in purple sweet potato^32^,^33^ were analyzed by HPLC-MS-MS (Supplementary Fig. S2). The content of these flavonols was significantly increased in the leaves, stems and developing storage roots (S16) of the *Lc* transgenic lines compared to the WT (Fig. 2B). The total flavonol content (quercetin-3-*O*-hexose-hexoside + quercetin-3-*O*-glucoside) in the leaf, stem and root of Lc1 was 83.8 μg/g, 6.89 μg/g and 2.91 μg/g, respectively, which is 13.05-fold, 6.75-fold and 4.62-fold higher than that in the WT, respectively. Similar changes were also observed in Lc2 and Lc3 (Fig. 2B). These results indicate that the biosynthesis of flavonols was increased in the *Lc* transgenic plants.

To further explore the effect of Lc on the flavonoid metabolic pathways, the expression of structural genes involved in the flavonoid pathway was analyzed. The expression of *Lc* in purple sweet potato led to an increase in the transcript levels of most flavonoid biosynthetic genes, which had different expression patterns in the leaves, stems and developing storage roots (S16) (Fig. 2C). In the leaves, the transcript levels of the genes *CHI*, *CHS, F3H, DFR, ANS, 3GT* and *FLS* were dramatically increased in the Lc lines compared to the WT. In particular, more than 100-fold change was observed in the transcript levels of the *DFR* gene. No significant difference was observed in the expression of the *PAL* gene (Fig. 2C, upper panel). In contrast, expression of the *PAL* gene as well as several other genes such as *CHS* was dramatically increased in the stems and roots of the *Lc* transgenic lines. Compared to the WT, the Lc lines showed a 4.33-fold and 4.87-fold change in the expression of *PAL* in the stems and developing storage roots (Fig. 2C, middle and bottom panels). These results imply that Lc regulates expression of the flavonoid biosynthetic genes differentially between leaves and stems/roots, which means that it possibly interacts differently with the other transcription factors in these organs. It cannot be excluded that the differential expression of the pathway genes could be due to involvement of tissue specific regulatory proteins that act coordinately with Lc through protein-protein interaction or concurrent binding of these proteins to their promoters. The bHLH factor-binding G-box (CACGTG) or E-box (CANNTG) motifs are present in the promoter regions of most flavonoid biosynthetic genes (Supplementary Fig. 3A). The results of a yeast-one-hybrid assay also indicated that Lc was able to bind to the G-boxes (CACGTG) of some of the sweet potato genes, such as *CHS*, *DFR*, and *ANS*; mutation of the G-boxes (C**TATA**G) resulted in the loss of its binding capacity (Supplementary Fig. 3B,C).

### *Lc* transgenic sweet potato showed retarded development of storage roots but no changes in the photosynthesis capacity of the leaves

**Figure 3:**
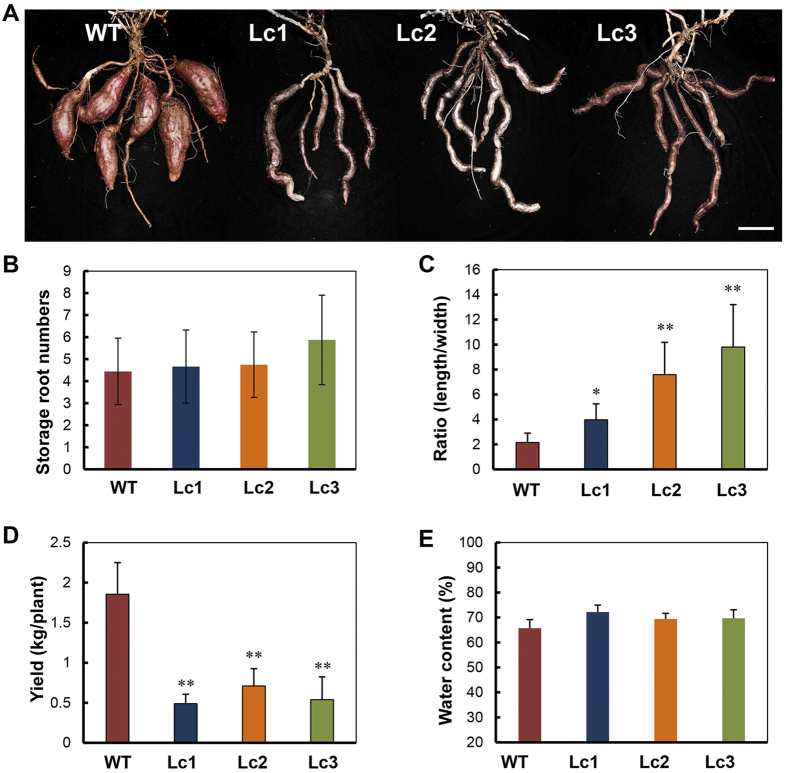
Morphological and physiological analyses of the mature storage roots (>S18) of 5-month-old wild-type and Lc transgenic sweet potato plants harvested from the field. (**A**–**C**) Shape (**A**), number per plant (**B**), length/width ratios (**C**) of the storage roots. WT, wild type; Lc1–3, independent *Lc* transgenic lines. (**D**,**E**) The yield (**D**) and water content (**E**) of the storage roots. Error bars represent the SE of three replicates. Asterisks indicate a significant difference compared to the WT at *P < 0.05 or **P < 0.01 (*t*-test). Bar = 5 cm.

After the plants were harvested from the field, clear phenotypic changes were observed in the mature storage roots of the 5-month-old Lc plant lines compared to the WT plants (Fig. 3A). Under field conditions, the WT plant produced 4–6 round-elliptical storage roots per plant with a short root stalk. The *Lc* transgenic lines produced similar numbers of storage roots per plant, but these storage roots were long and irregular or curved in shape and exhibited reduction in storage root ‘bulking’ or growth (Fig. 3A,B). The length/width ratio of the storage roots in the *Lc* transgenic lines was significantly increased, ranging from 3.98 (in Lc1) to 9.80 (in Lc3), much higher than that of the WT (2.15 in average, Fig. 3C). The per plant yield was remarkably decreased (0.49 kg in Lc1) compared to the WT (1.86 kg) (Fig. 3D). However, the water content was similar among the *Lc* transgenic lines and the WT (65%, Fig. 3E), indicating that the storage roots in all the plants were in the same developmental stages.

In order to identify the cause of the yield reduction, the photosynthesis capacity of the plants was measured. No change in the maximal quantum yield of PSII (Fv/Fm) was detected between the *Lc* transgenic lines and WT in leaves from different developmental stages (Supplementary Fig. 4). These results indicate that the increased anthocyanin content in the leaves of the *Lc* transgenic lines has no impact on the primary photochemistry of PSII. Thus, Lc might regulate other pathways/metabolisms to affect storage root growth.

### Lc up-regulates the lignin biosynthesis pathway and results in an increase in the lignin content of storage roots in sweet potato

**Figure 4:**
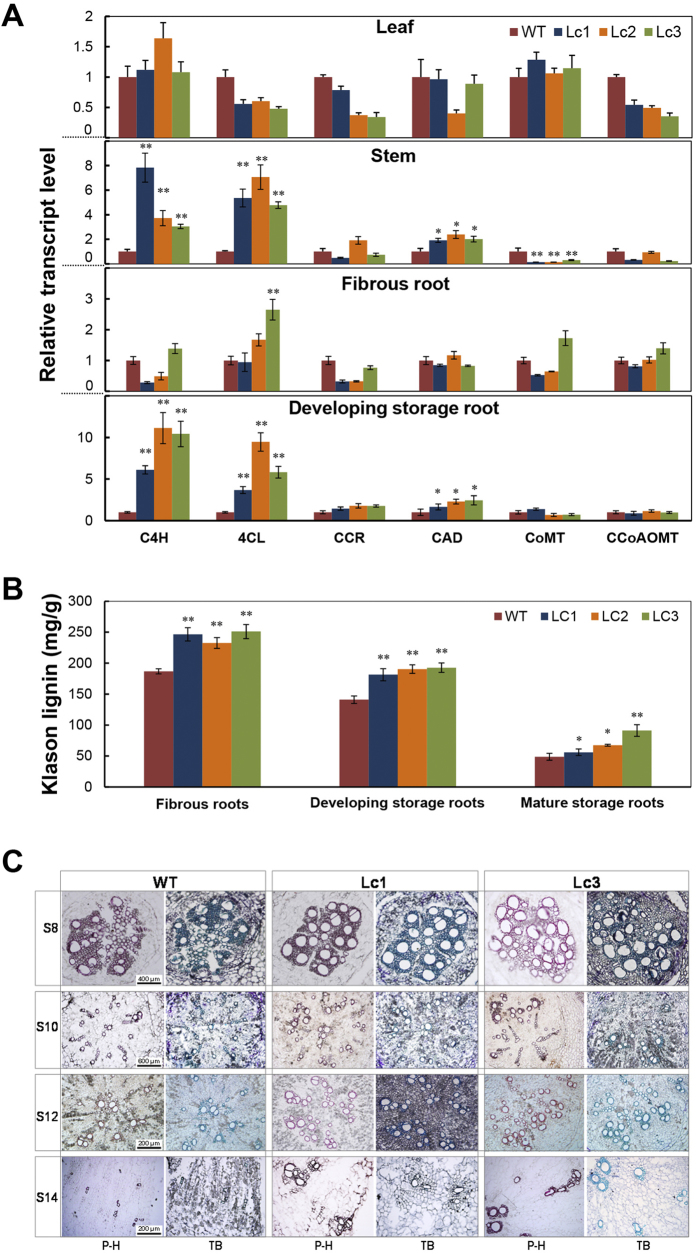
Effect of *Lc* expression on lignin biosynthesis and deposition in wild-type and *Lc* transgenic sweet potato. (**A**) qRT-PCR analysis of the changes in the transcript levels of major lignin biosynthetic genes in the leaves, stems, fibrous roots (S5-S8) and developing storage roots (S16) of 2-month-old wild-type (WT) and *Lc* transgenic sweet potato. Lc1–3, independent *Lc* transgenic lines. C4H, cinnamic acid 4-hydroxylase; 4CL, 4-coumarate-CoA ligase; CCoAOMT, caffeoyl-CoA *O*-methyltransferase; CCR, hydroxycinnamoyl-CoA reductase; CAD, cinnamyl alcohol dehydrogenase; COMT, caffeic acid/5-hydroxyferulic acid *O*-methyltransferase. (**B**) Klason lignin contents in the fibrous roots (S5-S8), developing storage roots (S10) and mature storage roots (>S18) of 5-month-old field-grown sweet potato plants. (**C**) Lignin deposition patterns in the initiating storage roots (S8), early developing storage roots (S10 and S12) and late developing storage roots (S14) of 1.5-month-old plants after staining with two dyes, phloroglucinol-HCl (P-H) and toluidine blue (TB). Error bars represent the SE of three independent replicates. Asterisks indicate a significant difference compared to the WT at *P < 0.05 or **P < 0.01 (*t*-test).

The up-regulation of *PAL* in the stem and storage roots of *Lc* transgenic sweet potato (Fig. 2C) indicates that other phenylpropanoid pathway genes might be impacted by overexpression of this regulatory protein. PAL gene promoter contains many G/E-box elements that probably bind to MYC transcriptions factors (Supplementary Fig. 3). PAL is involved not only in anthocyanin biosynthesis but also lignin biosynthesis. Therefore, we hypothesized that Lc considerably up-regulates lignin biosynthesis in the storage root of *Lc* transgenic lines. Indeed, expression of all tested lignin biosynthetic enzymes C4H, 4CL, and cinnamyl alcohol dehydrogenase (CAD) was all greatly increased in the stems and developing storage roots (S16) of the *Lc* transgenic lines (Fig. 4A). Compared to the WT, the three transgenic lines showed 6.1- to 11.1-fold and 3.7- to 9.4-fold changes in the expression of *C4H* and *4CL* respectively. Expression of the *CAD* gene was also slightly up-regulated in the root and stem of the *Lc* transgenic lines compared to the WT (Fig. 4A). The expression of these genes was unchanged in the leaves of *Lc* transgenic and WT plants. Further, the G/E-box motifs/elements could also be present in their upstream sequences (Supplementary Fig. 3A).

To further verify up-regulation of the lignin biosynthetic pathway in the Lc lines, the lignin level of fibrous roots (S5-S8), developing storage roots (S10) and mature storage roots (>S18) of field-grown plants was measured. Significant increase of Klason lignin was detected in Lc lines, by a minimum of 24.5% in fibrous roots and 28.6% in developing storage roots, compared to the WT value (Fig. 4B). In mature storage roots, the total Klason lignin content of Lc1, Lc2 and Lc3 ranged from 56.0 to 91.2 mg/g (DW), which was significantly higher than that of the WT (48.8 mg/g DW, Fig. 4B). Lc3 had the highest lignin content, 1.78 times that in the WT. Phloroglucinol-HCl or toluidine blue staining for lignin in the initiating, early and late stage developing storage roots (S8-S14, diameter < 2 cm) from plants 45 days after planting also revealed a higher number of lignified cells around xylem bundles in Lc1 and Lc3, as indicated by the increased coloration in comparison with the WT (Fig. 4C). Especially at the stage of S14, more lignified xylem elements and less starch granules were clearly noticeable in the Lc lines. There was no obviously change in lignin deposition patterns and levels in the stems between Lc lines and WT (Supplementary Fig. S5). These data showed that the lignification of developing storage roots in the *Lc* transgenic lines is elevated by up-regulation of the lignin biosynthesis pathway, which leads to reduced yield and altered shape of the storage roots.

### Starch metabolism in the storage roots of Lc sweet potato is promoted by starch degradation but not by starch synthesis

**Figure 5:**
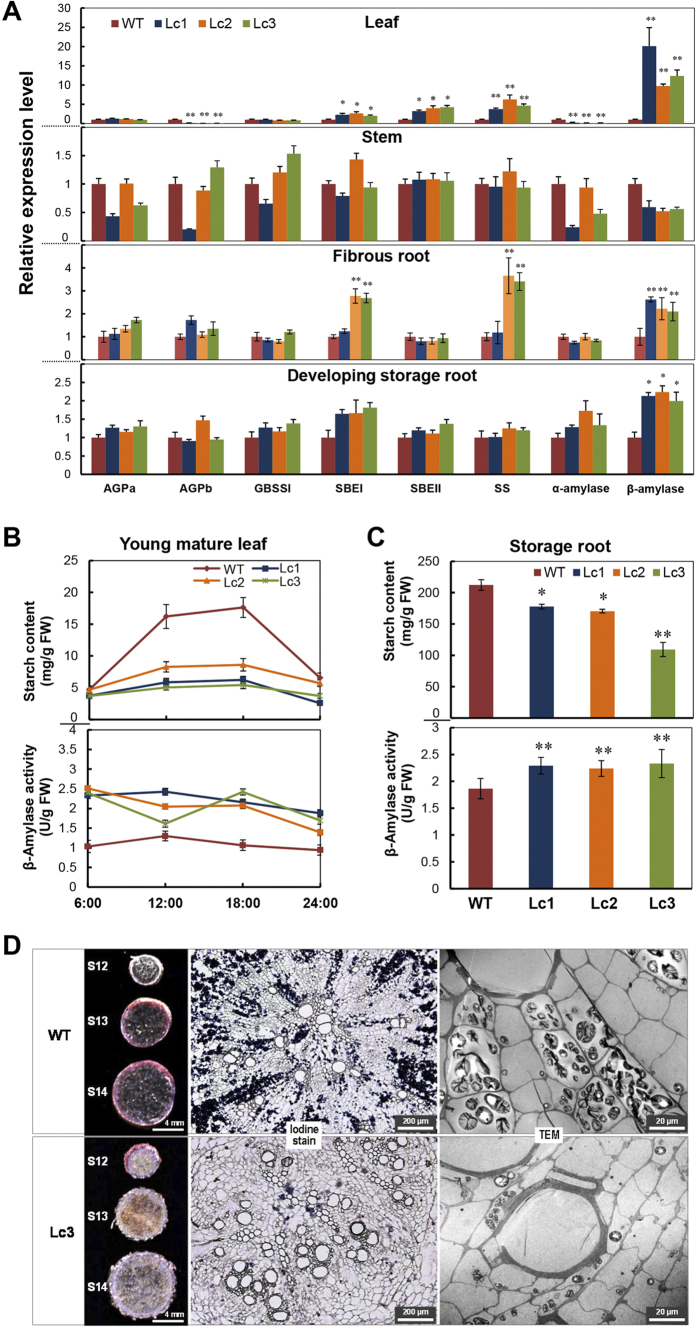
Starch metabolic gene expression profile, β-amylase activity and starch status in young mature leaves, storage roots of wild-type and *Lc* transgenic sweet potato. (**A**) Expression levels of the genes involved in starch biosynthesis (*AGPa*, *AGPb*, *GBSSI*, *SBEI*, *SBEII*, and *SS*) and starch degradation (α-amylase and β-amylase) in the leaf, stem and developing storage root (S16) of 2-month-old plants. WT, wild type; Lc1–3, independent *Lc* transgenic lines; AGPa, ADP-glucose pyrophosphorylase alpha subunit; AGPb, ADP-glucose pyrophosphorylase beta subunit; GBSSI, granule-bound starch synthase 1; SBEI, starch branching enzyme I; SBEII, starch branching enzyme II; SS, soluble starch synthase. (**B**) Starch content and β-amylase activity in response to diurnal rhythms in mature leaves. (**C**) Starch content in mature storage roots (S20) of field-grown plants and β-amylase activity of developing storage roots (S16). (**D**) Dark blue starch granules on sections of early developing storage roots (S12) of 2-month-old plants as determined using Lugol’s solution and TEM observation. Error bars represent the SE of three independent replicates. The asterisks indicate a significant difference compared to the WT at *P < 0.05 or **P < 0.01 (*t*-test).

Storage root development is strongly associated with starch accumulation. To confirm whether starch metabolism is affected by the expression of *Lc* in sweet potato, the expression profile of genes encoding for key enzymes involved in starch biosynthesis (AGPa, AGPb, SBEI, SBEII, SS, and GBBS1) and starch degradation (α-amylase and β-amylase) was analyzed by qRT-PCR. In the 2-months-old developing storage roots (S16), no changes were detected in the expression of *AGPa*, *AGPb*, *SBEI*, *SBEII*, *SS*, and *GBBS1* as well as the α-amylase gene. Only the β-amylase gene showed significant up-regulation (Fig. 5A, bottom panel). Similar observation was also found in fibrous roots. In the leaves, however, besides the β-amylase gene, the expression of *SBEI*, *SBEI* and *SS* was also increased by up to 5-fold compared to the WT (Fig. 5A, upper panel). β-amylase expression increased by 20-fold in the leaves and by 2-fold in the storage roots in Lc1, which raises the possibility that starch degradation is significantly enhanced for carbohydrate mobilization both in the source and the sink.

With regard to the diurnal rhythms of starch accumulation in leaves, the level of starch accumulation in Lc plants was lower than that in the WT during the entire day cycle and the first half of the dark cycle (Fig. 5B, upper panel). For example, at the end of the day cycle, the starch content in the WT leaves reached 17.6 mg/g fresh weight (FW), which is 2-folds that of the *Lc* transgenic plants. Consistent with this, the β-amylase activity in the leaves of *Lc* transgenic plants was always higher than that of the WT plants (Fig. 5B, lower panel). The average β-amylase activity was 2.41 ± 0.09 U/g at the end of the dark cycle (06:00) and 1.65 ± 0.25 U/g in the middle of the dark cycle (24:00) in the Lc lines; these values were much higher than that of the WT plants (1.03 U/g and 0.94 U/g, respectively; Fig. 5B). The starch content in the mature storage roots (S20) of the *Lc* transgenic plants was significantly lower than that in the WT (Fig. 5C, upper panel), and the β-amylase activity was significantly higher (average, 2.29 ± 0.05 U/g), about 23.1% higher than that in the WT (Fig. 5C, lower panel). Iodine staining and TEM observation of the early developing storage root (S12) in the WT plants 45 days after planting revealed the presence of dark-blue starch granules along the xylem rays (Fig. 5D, upper panel). However, clear starch granule clusters (Fig. 5D, lower panel) were not found in the Lc3 transgenic plants, which indicates that starch accumulation was repressed in this transgenic line. Upon the iodine staining of early developing storage roots (S12 to S14), the reduced coloration of dark blue further confirmed the observation (Fig. 5D). Together with the previous lignin assays, these results suggest that lignification competes with starch accumulation for the distribution of photo-assimilates in developing storage roots.

### The leaves and storage roots of Lc sweet potato have reduced sugar content

**Figure 6:**
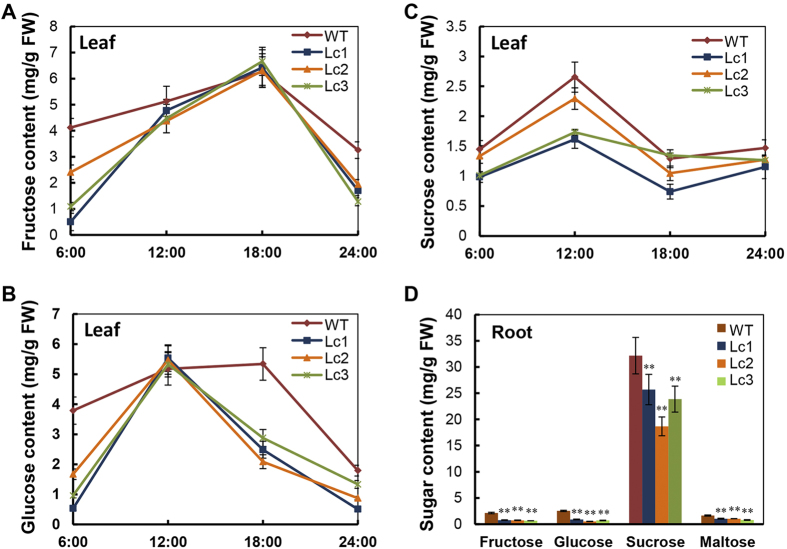
Sugar content changes in the leaves in response to diurnal rhythms and in the mature storage roots (>S18) of wild-type and Lc transgenic lines. (**A**–**C**) Changes in the content of fructose (**A**), glucose (**B**) and sucrose (**C**) at 6:00, 12:00, 18:00 and 24:00. WT, wild type; Lc1–3, independent *Lc* transgenic lines. (**D**) The content of individual sugars in the mature storage roots. Error bars represent the SE of three independent replicates. The asterisks indicate a significant difference compared to the WT at *P < 0.05 or **P < 0.01 (*t*-test).

As indicated previously, the expression and enzymatic activity of β-amylase were all up-regulated in the leaves and storage roots of *Lc* transgenic plants (Fig. 5A,C). In line with this, the fructose and glucose contents in the leaves of *Lc* transgenic plants were lower compared to the WT in the dark cycle. In the Lc plants, fructose levels were similar to those of the WT from 12:00 to 18:00, while glucose levels were similar at 12:00 and decreased significantly thereafter (Fig. 6A,B). The sucrose content in the WT leaves seemed to be always slightly higher than that of *Lc* transgenic plant leaves (Fig. 6C). The fructose, glucose, sucrose, and maltose contents in the mature storage roots (S20) of the *Lc* transgenic lines were lower than that of the WT. The sucrose content (average, 22.7 ± 3.7 mg/g) in the Lc lines was 30% lower than that in the WT. Similarly, the fructose, glucose and maltose levels were also reduced in the storage roots of the Lc plant lines, by 66.5%, 72.1% and 42.4%, respectively, in comparison with the WT (Fig. 6D). These results suggest that lignification results in greater carbon flux and may reduce the sink strength for starch accumulation in *Lc* transgenic sweet potato.

## Discussion

The development of storage roots—from the anatomical features to the molecular regulation mechanisms—has received attention of the research community for a long time^2^,^3^,^11^. The fibrous roots, pencil roots and storage roots, which comprise the sweet potato root system, are distinguishable from each other by their shape and histochemical structures^2^. The promotion of storage root formation and inhibition of pencil root production with proper field management is necessary for ensuring a high yield in sweet potato cultivation^12^,^13^,^34^,^35^. The development of the pencil root, which is a type of stele lignified thick root, is an irreversible process that is affected by various environmental and regulatory factors^14^. Storage root development is strongly associated with the down-regulation of lignin biosynthesis and up-regulation of starch biosynthesis^11^; it has been suggested that the carbon-flux distribution across the starch and lignin metabolic pathways influences the development of fibrous roots towards pencil roots or storage roots. In our study, *Lc* transgenic purple sweet potato showed up-regulated lignin biosynthesis, in addition to flavonoid biosynthesis (including anthocyanins and flavonols), which resulted in a significant increase in lignification during the early stages of storage root formation; these processes led to elongated shape of the storage root, reduced root size and yield, and was correlated with less starch accumulation. Our findings indicate that the shift in carbon flux towards lignin biosynthesis is associated with increased starch degradation both in the sink and source, and retarded starch accumulation in the parenchymal cells of the developing storage roots.

Increased lignin content of purple sweet potato expressing the maize Lc transcription factor is a suitable model for studying the relationship between primary metabolism and secondary metabolism in storage root development. Many studies have shown that Lc promotes anthocyanin biosynthesis and hence anthocyanin pigmentation and production of flavonoids of other classes in various plant species^27^,^28^,^29^,^36^,^37^,^38^, but the commitment to lignin deposition or other functions has seldom been reported^39^. In *Lc*-expressing transgenic tomato and petunia plants^27^,^28^, the level of *PAL* transcripts did not differ significantly across different tissues, but the level of *PAL* transcripts was up-regulated in apple^29^. No other genes related to lignin biosynthesis have been studied in *Lc*-overexpressing plants^27^,^29^. In our *Lc* transgenic sweet potato, besides *PAL*, other genes of the lignin biosynthesis pathway such as *C4H*, *4CL* and *CAD* were significantly up-regulated in stems and storage roots, but no changes in their expression were found in leaves. Moreover, increased lignification was observed during early storage root formation and increased lignin content in the field-harvested storage roots. Therefore, Lc-mediated up-regulation of lignin biosynthesis in purple sweet potato appears to be organ-specific, which is different from the case with Lc-mediated regulation of anthocyanin accumulation. This indicates that other regulatory factors interact with Lc directly or indirectly to control lignin deposition in different tissues^40^. In addition, the increased accumulation of flavonols (quercetin-3-*O*-hexose-hexoside and quercetin-3-*O*-hexoside) in leaf, stem and root of Lc lines is correlated with the upregulation of *FLS* expression (Fig. 2B,C).

As a major branching point between the primary and secondary metabolic pathways in plants, PAL directs up to 30% of the fixed carbon source from the Shikimate pathway to the phenylpropanoid metabolic pathways^15^,^41^,^42^,^43^,^44^. Since up-regulation of starch biosynthesis and down-regulation of lignin biosynthesis are associated with storage root development^11^, and starch accumulation occurs even during the initiation stage, and during the early bulking stage of storage roots, enhanced lignin deposition may greatly influence starch accumulation and decrease the starch content in field-harvested storage roots. Indeed, accumulation of starch granules was dramatically reduced in early developing storage roots of *Lc* transgenic sweet potato according to the findings of the iodine-staining assay; however, this reduction was probably not due to changes in the starch biosynthesis capacity, as indicated by the unchanged expression of AGPase, SBE, SS and GBBS1. Furthermore, starch degradation was found to be augmented both in leaves and storage roots, which indicates that starch mobilization was promoted and can explain the reduced transient starch accumulation in leaves during the day cycle. The significant reduction in sugar levels in developing storage roots and leaves also confirms that the developing storage roots of *Lc* transgenic sweet potato have altered sink capacity for driving the distribution of photo-assimilates towards lignin deposition.

Reduction in shoot growth has been reported in apple, tomato, petunia hybrids, bentgrass and Arabidopsis plants that heterologously express the *Lc* gene^28^,^29^,^30^,^45^. These findings suggest that high levels of anthocyanin accumulation might have detrimental effect on the growth and development of these plants since the flavonoids can act as auxin transport inhibitors to negatively regulate polar auxin transport *in vivo* and disturb the transport of endogenous auxins^46^,^47^,^48^. Auxin retention by flavonoid action may also affect vascular differentiation^49^. In our *Lc*-expressing sweet potato, the reduced storage root size and yield seem not to be related with auxins, since the expression levels of the polar auxin transport genes *AUX1*, *PIN1a* and *PIN1b* were unchanged (Supplementary Fig. 6). The photosynthesis capacity was also unaffected. Undoubtedly, the interaction between Lc and native transcription factors related to the regulation of starch and phenylpropanoid metabolism in sweet potato will provide insights into the regulation of storage root development. For example, down-regulated expression of the transcription factor SRD1, which is essential for propagation of metaxylem in sweet potato, showed increased lignin biosynthesis in storage roots^19^. Using the *Lc* transgenic sweet potato to study the changes of regulatory component might eventually unveil the molecular links between lignification and starch accumulation in developing storage roots.

**Figure 7:**
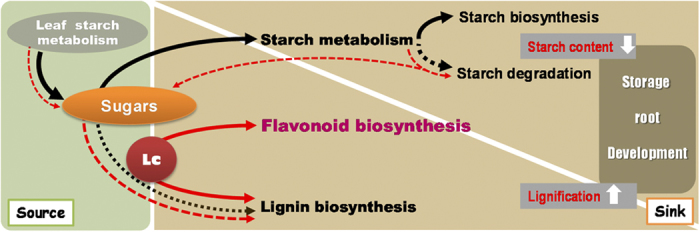
Schematic illustration of the intrinsic relationship between phenylpropanoid and starch metabolisms with regard to the regulation of storage root development in sweet potato mediated by the maize Lc transcription factor. Normally, the photo-assimilates (sugars) from leaves are transported to roots for the use of starch biosynthesis (black arrows) to support storage root development; during the process, lignin biosynthesis as well as starch degradation is down-regulated (dashed black arrows). Up-regulation of flavonoid and lignin biosynthesis by Lc increases flavonoid accumulation and lignin deposition in storage roots at the initiation stage (red arrows), which results in increased carbon flux by the mobilization of starch degradation both in the roots (sink) and leaves (source)(dashed red arrows). As a result, starchy storage root development is impacted.

Based on the findings of our study, a molecular model depicting the regulation of storage root development, by the transcription factor Lc, was proposed (Fig. 7). Normally, during the initiation of storage roots, the carbon flux derived from the channeling of photo-assilimates (sugars) towards starch biosynthesis is increased. Simultaneously, lignin biosynthesis is repressed. The expression of Lc in sweet potato causes not only greater flavonoid accumulation but also enhanced lignin biosynthesis, leading to lignification in the initiating storage roots. This process triggers an increase in the carbon flux towards phenylpropanoid metabolisms: more photo-assimilates are produced via degradation of transient starch from the source (leaf) and storage starch from the sink (the storage roots) by β-amylase for partitioning into the sink for lignin deposition. Finally, transgenic sweet potato shows smaller size and yield of the storage roots.

In conclusion, ectopic expression of Lc in purple sweet potato up-regulates the phenylpropanoid biosynthesis pathway, which favors flavonoid accumulation and lignin deposition; this directly affects starch metabolism by causing an increase in starch degradation. The storage roots of *Lc* transgenic sweet potato showed altered shape and reduced size and yield. This provides evidence of how the carbohydrate regulatory mechanism affects sweet potato storage root development as well as increases our understanding of the function of transcription factors in regulating primary and secondary metabolism. Nevertheless, there are questions that are yet to be answered, such as how endogenous transcription factors regulate the developmental process and partitioning of photo-assimilates.

## Methods

### Plant material

The purple-fleshed sweet potato (*Ipomoea batatas* Lam.) cultivar Ayamurasaki, developed by Kyushu National Agricultural Experiment Station^50^, was used as a donor for genetic transformation. The cultivar has high anthocyanin content in its storage roots with deep purple skin. Its shoots have green and lobed mature leaves. Untransformed and transgenic plants subcultured from *in vitro* plantlet cultures were transferred into plastic pots (18 cm in diameter) containing dark soil and vermiculite in a ratio of 2:1 (v/v) and grown in a growth chamber under a 16-h light/8-h dark photoperiod at 25 ± 3 °C. One-month-old shoots were transplanted into the field in early May for evaluation of the phenotype and agronomic traits with a 5-month growth period at the Wushe experimental station, Songjiang, Shanghai. To better classify the storage root stages, the developmental process from fibrous root to mature storage roots are divided into 20 stages including Fibrous Root (maximum diameter < 2 mm, S1-S8), Initiating Storage Root (2 mm < maximum diameter < 5 mm, S9-S13), Developing Storage Root (5 mm < maximum diameter < 20 mm, S14-S17) and Mature Storage Root (maximum diameter > 5 mm, S18 to S20). The lignified thick pencil roots cover the stages from S9 to S13 in size (Fig. 1A).

### Plasmid and *Agrobacterium*-mediated sweet potato transformation

The expression cassette of the maize *Lc* gene (Genbank accession No. M26227.1) driven by the cauliflower mosaic virus promoter 35S (*CaMV* 35S) was cloned into pCAMBIA1300 to generate the binary vector p35S-Lc. Genetic transformation of sweet potato was carried out according to a previously described method by Yang *et al.* (2011)^31^. Briefly, embryogenic calli induced from the shoot meristem tissues of the cultivar Ayamurasaki were used for establishment of embryogenic suspension cultures. Then, the 1-month-old suspension culture was used as the explant for cocultivation with *Agrobacterium tumefaciens* strain LBA4404 harboring the binary vector p35S-Lc. The transformed calli were selected after culturing on a plant regeneration medium containing 10 mg/l hygromycin for 1 month and then transferred onto fresh medium for plant regeneration and growth^31^.

### Molecular and phenotypic characterization of the transgenic plants

Genomic DNA was isolated from the leaves of greenhouse-grown WT and PCR-positive transgenic plants as described by Kim and Hamada (2005)^51^. For Southern blot analysis, 15–20 μg of genomic DNA was digested with *Hin*dIII at 37 °C overnight and separated by electrophoresis on a 0.8% agarose gel overnight; the gels were blotted on a positively charged Amersham Hybond N^+^ nylon membrane (GE Healthcare, Life Sciences, Indianapolis, USA) for hybridization with the DIG-labeled PCR product of the transgenes. For establishing the hygromycin phosphotransferase gene (*HPT*) probe, 100 pg of plasmid was used as the template to amplify a 500-bp fragment with a PCR DIG Probe Synthesis Kit (Roche Applied Science, Manheim, Germany) using the following primer pairs: 5′-TTCTACACAGCCATCGGTCC-3′ (forward) and 5′-CCCATGTGTATCACTGGCAA-3′ (reverse). Hybridization and detection were performed according to the manufacturer’s instructions, using the DIG-High Prime DNA Labeling and Detection Starter Kit II (Roche Diagnostics, Manheim, Germany).

The expression of *Lc* mRNA was analyzed by reverse transcriptase PCR (RT-PCR). Total RNA was extracted from the developing storage root (S16), stem and leaf tissues of 2-month-old greenhouse-grown plants using the RNAprep Pure Plant kit (Tiangen, Beijing, China) following the manufacturer’s instructions. The total RNA was digested with DNase at 37 °C for 15 min and then reverse transcribed into cDNA using M-MLV Reverse Transcriptase RNaseH (Toyobo, Osaka, Japan). For RT-PCR analysis, the *Lc* primers (forward, 5′-ATGGCGCTTTCAGCTTCCCG-3′; reverse, 5′-TGGACGCGCTCTTGGCCAGG-3′) were used to amplify a 520-bp product, and the actin gene primers (forward, 5′-CTGGTGTTATGGTTGGGATGG-3′; reverse, 5′-GGGGTGCCTCGGTAAGAAG-3′) were used as the internal control. The transcript levels of the *Lc* gene in the transgenic lines were further analyzed by quantitative RT-PCR (qRT-PCR) using the following primers: 5′-ACGGGAGCAGCACAGGAAAT-3′ (forward) and 5′-CGACGCTTTGTTCACCCTGT-3′ (reverse).

The phenotype of the *Lc* transgenic and WT sweet potato was studied under the greenhouse and field conditions. The growth features of the above-ground plant shoots, the shape and color of the leaves and stems, the shape and size of the storage roots, and the color of a cross-section of the storage root (S18) were recorded. The yield was the average weight of mature storage roots of five 5-month-old plants per line.

### Expression analysis of key genes involved in the flavonoid, lignin and starch metabolism pathways

The transcript level of the biosynthesis genes involved in the flavonoid (*PAL*, *CHI*, *CHS*, *F3H*, *FLS*, *DFR*, *ANS* and *3GT*), lignin (*C4H*, *4CL*, *CCR*, *CAD*, *CoMT* and *CCoAOMT*) and starch (*AGPa*, *AGPb*, *GBSSI*, *SBEI*, *SBEII*, *SS*, α-amylase and β-amylase) metabolism pathways was analyzed by qRT-PCR. Total RNA extraction from the 3^rd^ leaf, stem, fibrous root and the developing storage root (S16) of greenhouse-grown plants, and cDNA synthesis were carried out as previously described in this section. The expression of the genes involved in the metabolic pathways was analyzed using qRT-PCR with the SYBR green fluorescent dye in a Bio-Rad CFX96 thermocycler (Bio-Rad, Hercules, CA, USA). The qRT-PCR cycling parameters were as follows: initial denaturation at 95 °C for 1 min; 40 cycles at 95 °C for 20 s, 60 °C for 20 s and 72 °C for 20 s; and a final extension step at 72 °C for 5 min. The qRT-PCR primers used to amplify the genes (Table S1) were designed using the Primer3.0Plus software (http://www.bioinformatics.nl/cgi-bin/primer3plus/primer3plus.cgi) according to the EST database for sweet potato at our laboratory. The gene expression level was calculated using the comparative Ct method^52^.

### Analysis of flavonoid and lignin biosynthesis gene promoters and yeast one hybrid assay

The upstream genomic sequences of the anthocyanin biosynthesis genes (*CHI, CHS, F3H, DFR, ANS* and *3GT*) and lignin biosynthesis genes (*PAL, C4H, 4CL, CCR, CAD, COMT* and *CCoAOMT*) were searched in public genome databases (e.g. Genbank) and our own database. The available promoter fragments were analyzed for the cis-element using the PlantCARE datebase (http://bioinformatics.psb.ugent.be/webtools/plantcare/html/) and PLACE (http://www.dna.affrc.go.jp/PLACE/). The G-box (5′-ATCACGTGCT-3′) present in these promoters was verified for the binding capacity with Lc. Briefly, for DNA binding analysis, the open reading frame of *Lc* was amplified by PCR using the primer pair 5′-GCCGAATTCATGGCGCTTTCAGCTTC-3′ (forward) and 5′-GCCGTCGACTCACCGCTTCCCTATAGCT-3′ (reverse), and was cloned into pGAD424 to construct pGAD424-Lc. The plasmid was then transformed into the yeast strain YM4271, which was co-transformed with the reporter vector pHIS-G-BOX containing three copies of the G-box to determine the interaction between Lc and G-BOX. The pHIS-mG-BOX (5′-ATC**TATA**GCT-3′) containing the mutated G-box with a 4-bp substitution at the center was used as a negative control. The analysis was carried out according to the manufacturer’s specifications (Clontech, Palo Alto, CA, USA).

### Extraction and quantification of anthocyanins and flavonols

The total anthocyanins in the WT and *Lc* transgenic plants grown in the greenhouse were extracted using a previously described method^53^. Briefly, approximately 100 mg of lyophilized fully expanded 3^rd^ leaves from the top, 100 mg of lyophilized young stem and 500 mg of lyophilized developing storage roots (S16) were extracted twice with 10 mL of 5% formic acid to fully dissolve the anthocyanins. The suspensions were centrifuged at 4,000 rpm for 10 min, and the supernatants were filtered through a 0.22-μm nylon filter. To determine the total anthocyanin content, absorbance was read at 530 nm using a spectrophotometer (DU730UV VIS; Beckman Coulter, USA). The standard curve constructed from the cyanidin 3-*O*-sophoroside concentrations was also measured at OD_530_. The total anthocyanin content in the WT and *Lc* transgenic lines was quantified as cyanidin 3-*O*-sophoroside equivalents.

The total flavonols in the greenhouse-grown WT and *Lc* transgenic lines were extracted and analyzed by HPLC mass spectrometry using previously described methods with slight modifications^54^,^55^. Briefly, approximately 200 mg of young leaves, 200 mg of stem and 500 mg of developing storage roots (S16) were extracted twice with 5 mL (leaf and stem) or 7 mL (storage root) of acetone/water/acetic acid (70:29.5:0.5, v/v/v). After centrifuged at 4,000 rpm for 10 min, the supernatant was filtered through a 0.22-μm nylon filter before it was used for further analysis. Flavonol analysis was carried out according to Wang *et al.* (2013) with the HPLC1200-MSD/Q-TOF 6520 system (Agilent, Waldbronn, Germany) on an electrospray ionization (ESI) source, which has dual nebulizers to allow the reference mass to be corrected prior to monitoring^33^. For HPLC separation, a C18 reverse-phase column (Agilent ZORBAX Eclipse XDB; 4.6 × 50 mm; 1.8 μm) was connected to an autosampler and washed at a flow rate of 0.2 mL/min. The mobile phase solvents were composed of 0.5% (v/v) acetic acid in water (solvent A) and acetonitrile (solvent B). The flavonols were quantified using external calibration curves of the quercetin-3-*O*-glucoside standard. The flavonol concentration was determined in triplicate.

### Lignin content measurement and lignin deposition by histochemical staining of storage roots

Analysis of the Klason lignin content was performed with the sulfuric acid digestion method^56^,^57^, using fibrous roots (S5-S8), developing storage roots (S10) and mature storage roots (>S18) harvested from the field. In short, 1 g (W_1_) dry weight of the storage root of sweet potato was fully digested with 12 M H_2_SO_4_ at 30 °C overnight and then diluted with water in 0.4 M H_2_SO_4_. The sample was autoclaved at 121 °C for 60 min. After washing with hot water until the H_2_SO_4_ residue was eliminated, the non-hydrolyzed sample was collected by filtration through a fibertec P2 crucible and dried for 2 h at 130 °C. After cooling, the sample (W_2_) was weighed in a desiccator. Finally, the sample was ashed at 525 °C for about 3 h in a muffle furnace and the cool residue left in the desiccator was weighed (W_3_) again. The Klason lignin content was calculated using the following formula: Klason lignin (%) = (W_2_ − W_3_) × 100/W_1_.

Cross-sections of the initiating roots (S8), early developing storage root (S10 and S12) and late developing storage roots (S14) harvested from 1.5-month-old plants in the greenhouse were fixed in 4% neutral-buffered formalin for 24 h and then embedded in paraffin wax. The 15-μm thick sections were cut and placed on silane-coated slides to fix the samples. After drying overnight at 37 °C, the sections were dewaxed, rehydrated and stained with phloroglucinol-HCl (one volume of concentrated HCl was mixed with two volumes of 0.5% phloroglucinol in ethanol) or toluidine blue (0.05%). After incubation with phloroglucinol-HCl for 10 min or toluidine blue for 3 min at room temperature, the samples were rinsed with water to get rid of the staining solution until the wash solution was clear. The lignin deposition patterns were observed under an Olympus BX51 microscope with the Olympus DP controller software (Olympus, Japan).

### Starch content analysis and iodine staining of storage roots

The total starch content of leaves and mature storage roots (>S18) from 5-month-old field-grown WT and transgenic sweet potato was determined using the Megazyme kit (Megazyme International Ireland Ltd. Co., Wicklow, Ireland) with slight modifications. Since the kit measures the reducing sugar content using a spectrophotometer at a wavelength of 510 nm, the measurement can be affected by the presence of anthocyanins that have the same absorbance wavelengths at 510 nm; therefore, the method was modified by using HPLC instead of measuring the absorbance wavelength at 510 nm. The amount of starch present in the leaf (100 mg) and storage root (100 mg) was determined from the pellets obtained after the soluble sugars were extracted. In brief, the pellet was suspended in 0.2 mL of aqueous ethanol (80% v/v). After the tubes were shaken on a vortex, 3 mL of thermostable α-amylase solution (100 U/mL) was immediately added. The tubes were incubated in a boiling water bath for 6 min by vigorous stirring every 2 min. Then, the tubes were placed in a bath at 50 °C and 100 μL amyloglucosidase (330 U) was added with stirring on a vortex mixer before incubation at 50 °C for 30 min. The final volume was adjusted to 10 mL with distilled water, after which the tubes were centrifuged at 3,000 rpm for 10 min. The glucose content of the supernatants was determined by the HPLC method using Agilent ZORBAX Carbohydrate Column (Agilent Technologies, 4.6 × 150 mm, 5 μm) according to the manufacturer’s protocol. The total starch content was determined from the glucose content according to the following formula: Starch content (mg/g FW) = glucose content × 162/180 (adjustment from free D-glucose to anhydro-D-glucose, as occurs in starch).

To investigate starch accumulation in storage roots, sections of the early developing storage roots (S12) of WT and transgenic plants of the same size were immersed in iodine staining solution (3.75 g KI and 1.25 g I_2_ in 500 mL of distilled water) for 10 min at room temperature. After washing with distilled water, the sections were observed under an Olympus BX51 microscope and imaged with the Olympus DP controller software (Olympus, Japan).

### Measurement of water and sugar content

Five-month-old *Lc* transgenic lines and WT plants were harvested, and the FW of the mature storage roots was measured. Chips of the fresh storage roots were dried at 80 °C for two days until a constant dry weight (DW) was attained. The water content in the storage roots was calculated using the following formula: water content (%) = (FW − DW)/FW × 100%.

Analyses and quantification of glucose, fructose, sucrose and maltose were performed as previously described with slight modifications^58^. Briefly, 100 mg of fresh leaves and storage roots ground in liquid nitrogen was dissolved in 0.7 mL of 80% methanol to extract the sugars. The sample was thoroughly vortexed and incubated for 45 min at 70 °C. Aliquots of 0.7 mL of HPLC-grade water and 0.7 mL chloroform were added to the sample. After shaking several times, the mixtures were centrifuged at 12000 *g* for 10 min. Then, 0.7 mL of the aqueous supernatant was transferred into 1.5-mL Eppendorf tubes and resuspended in 0.7 mL of chloroform. After centrifugation at 12000 *g* for 10 min, 0.5 mL of the supernatant was transferred to a glass tube for HPLC analysis of each sugar component. The sugar-separation method used was that described by the manufacturer but with slight modifications; the Agilent technologies HPLC column (ZORBAX Carbohydrate; 4.6 × 150 mm, 5 μm) with a differential refraction detector was used. The mobile phase consisted of 75% acetonitrile with a flow rate of 0.8 mL/min; the temperature of the column was maintained at 45 °C. The sugars were identified based on the retention time of the standards, and sample concentrations were calculated from the external standard curve.

### Measurement of β-amylase activity

β-Amylase activity in the greenhouse-grown leaves and developing storage roots (S16) of sweet potato was determined by the Betamyl-3^®^ method using commercial kits (Megazyme International Ireland Ltd. Co., Wicklow, Ireland), according to the manufacturer’s instructions. One gram of the ground fresh material was used for analysis. The β-amylase present in the samples hydrolyzes the substrate *p*-nitrophenyl-β-D-maltotrioside (PNPβ-G3) in the presence of thermostable β-glucosidase and stabilizers to produce maltose and *p*-nitrophenyl-β-D-glucose, which then immediately release d-glucose and *p*-nitrophenol in the presence of β-glucosidase. The liberated *p*-nitrophenol in the amylase assays was detected spectrophotometrically at 400 nm by a DU730UV VIS spectrophotometer (Beckman Coulter, Indianapolis, IN, USA).

### Determination of the photosynthetic activity of the leaves

Chlorophyll fluorescence was determined in young, mature and old leaves of the purple sweet potato collected from the greenhouse, using a chlorophyll fluorometer (Walz Imaging PAM; Walz GmbH, Effeltrich, Germany) after dark adaptation for about 30 min, according to the manufacturer’s instructions and previous reports^59^. Photosynthetic activity was recorded via chlorophyll fluorescence measurement of the Fv/Fm photochemical yield, which indicated the maximum quantum yield of PSII. The analysis was carried out at room temperature (25 °C) using saturated light flashes.

### Statistical analysis

All data are presented as mean ± SD from at least three independent experiments with three replicates each. The statistical significance of the differences was determined using the Student’s *t*-test. Differences between treatments were considered significant when P < 0.05 or 0.01 in a two-tailed analysis.

## Additional Information

**How to cite this article**: Wang, H. *et al.* Altered Phenylpropanoid Metabolism in the Maize *Lc*-Expressed Sweet Potato (*Ipomoea batatas*) Affects Storage Root Development. *Sci. Rep.* **6**, 18645; doi: 10.1038/srep18645 (2016).

## Supplementary Material

Supplementary Information
