# Supplementary material for: Altered Phenylpropanoid Metabolism in the Maize Lc-Expressed Sweet Potato (Ipomoea batatas) Affects Storage Root Development
Source: Sci Rep. 2016 Jan 4;6:18645. doi: 10.1038/srep18645 (PMC4698713; doi:10.1038/srep18645)
Supplement: Supplementary Information [file srep18645-s1.pdf]

Altered Phenylpropanoid Metabolism in the Maize *Lc*-Expressed Sweet Potato (*Ipomoea batatas*) Affects Storage Root Development. *Hongxia Wang, JunYang, Min Zhang, Weijuan Fan, Nurit Firon, Sitakanta Pattanaik, Ling Yuan and Peng Zhang*

SUPPLEMENTARY INFORMATION

Supplementary Table S1 | qRT-PCR primers for the genes related to the flavonoid, lignin and starch metabolism pathways

Primer	Forward primer (5'→3')	Reverse primer (5'→3')
qIbPAL	GGCGAGCACGAGAAGAATGT	ATGGCAGGGTTTCCGTTCTC
qIbCHI	GCCGAAGTCAAAGTGGAGAG	CGCCTATCGCCGTGAACTTG
qIbCHS	CGCACTTGGATAGCCTGGTC	ATCGGGAGCCAAGGTCTGCG
qIbF3H	CATCGTTTCCAGCCATCTCC	TTTCCGTTACTGCCCTCCAC
qIbFLS	CCTCCTTCTGCGGTGAACTA	CCTGCAGCTTCCTTCAACTC
qIbDFR	TTTATCGGCTCCTGGTTGGT	CGTGTCCGCTTTCGGTAGTT
qIbANS	GCGTCCCTAACTCCATCATC	AGAACACCGCCCAAGAAACC
qIbGT	CGCCCTAAAAGCCCCATT	CTCACAAAGCAGCCCACAGAT
qIbC4H	TGGTGATTTCATCCCCATTT	TTTTGCTGGGCTTCAAGAAT
qIb4CL	TATTTTCCGATCGAGGTTGC	ACTTTCCGGCAAATCAAATG
qIbCCR	GCAGAGATAACGGCCAGAAG	TTGCTACAACCCACCATCAA
qIbCAD	AGCTGGTAATGGTTGGCATC	TCCAAAGCCGTGTTGACATA
qIbCOMT	AAACGGGAAAGTGATCGTTG	CCATGATCCAAGTGTTGACG
qIbCCoAOMT	CCGGTTCTTGACCAGATGAT	TTCCACAGGGTGTTGTCGTA
qIbAGPa	TCGACGGTGATGTTAGCAAG	AACAGCCTTTGGAGAAACGA
qIbAGPb	GACAAGAACGTAAGGATTGGGA	CGAATGGTTGCTTTCTCCAT
qIbGBSSI	CAGTTGGTTTGCCAGTTGAC	ACGTTGAACTTTGCCACTCC
qIbSBEI	GGTTTACGGGTCTTGATGGA	AACAGCCTGCTATCCCACAC
qIbSBEII	CTTCCCTGAAGCCATAACCA	CCATTTGCCAATCCTCATCT
qIbSS	CGGTTCACTTTGCTTTGTCA	CATTGTGTGGGCGATACTTG
qIba-amlyase	CTGCATTTTTGTTCCTGCAA	TTCGATGCGTCCAAGTCATA
qIbB-amlyase	AGACTGGAAGGAGGCTGTGA	TGTTGGCTTCTTCGAGGACT
Ibactin	CTGGTGTTATGGTTGGGATGG	GGGGTGCCTCGGTAAGAAG

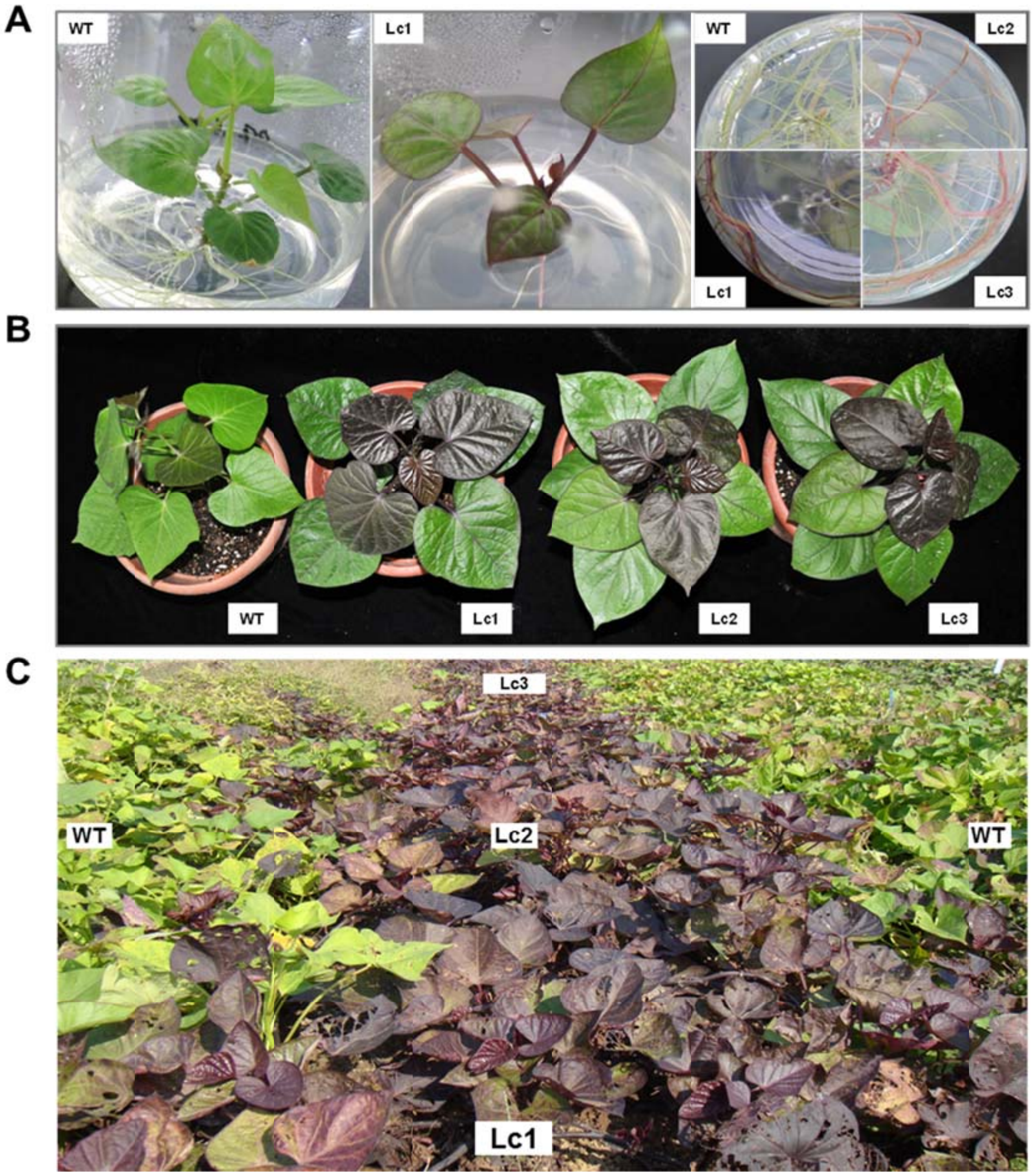

Supplementary Figure S1 | Enhanced pigmentation in Lc transgenic plants. (A) *In vitro* shoot culture. (B) Plantlets in the pots. (C) Plants in field before harvest.

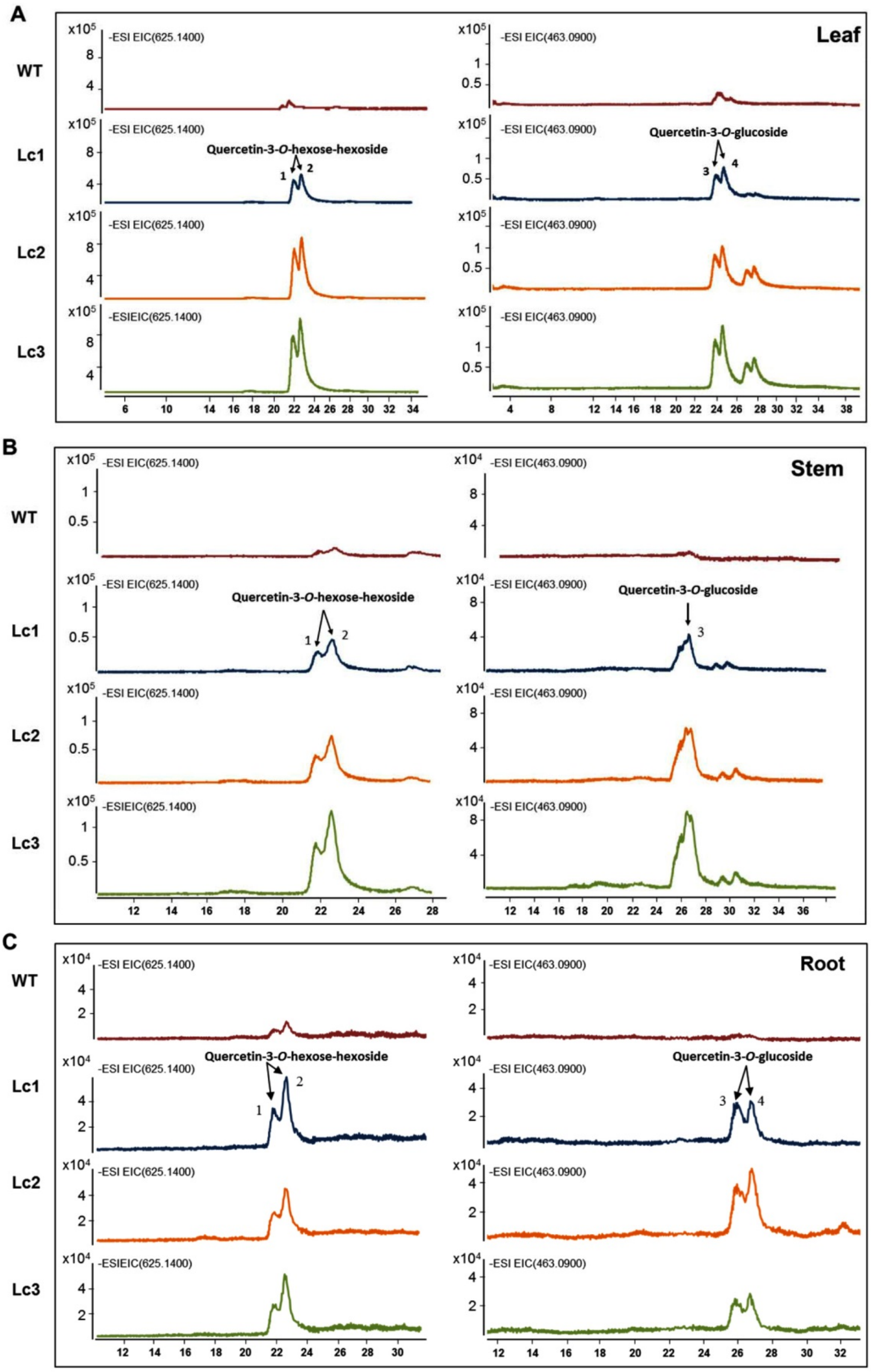

Supplementary Figure S2 | HPLC-MS analyses of flavonols in wild-type and *Lc* transgenic sweet potato. Leaf (A), stem (B) and developing storage root (C) were measured. Quercetin-3-O-hexose-hexoside (molecular weight, 625.14) and quercetin-3-o-glucoside (molecular weight, 463.09) are indicated by arrows.

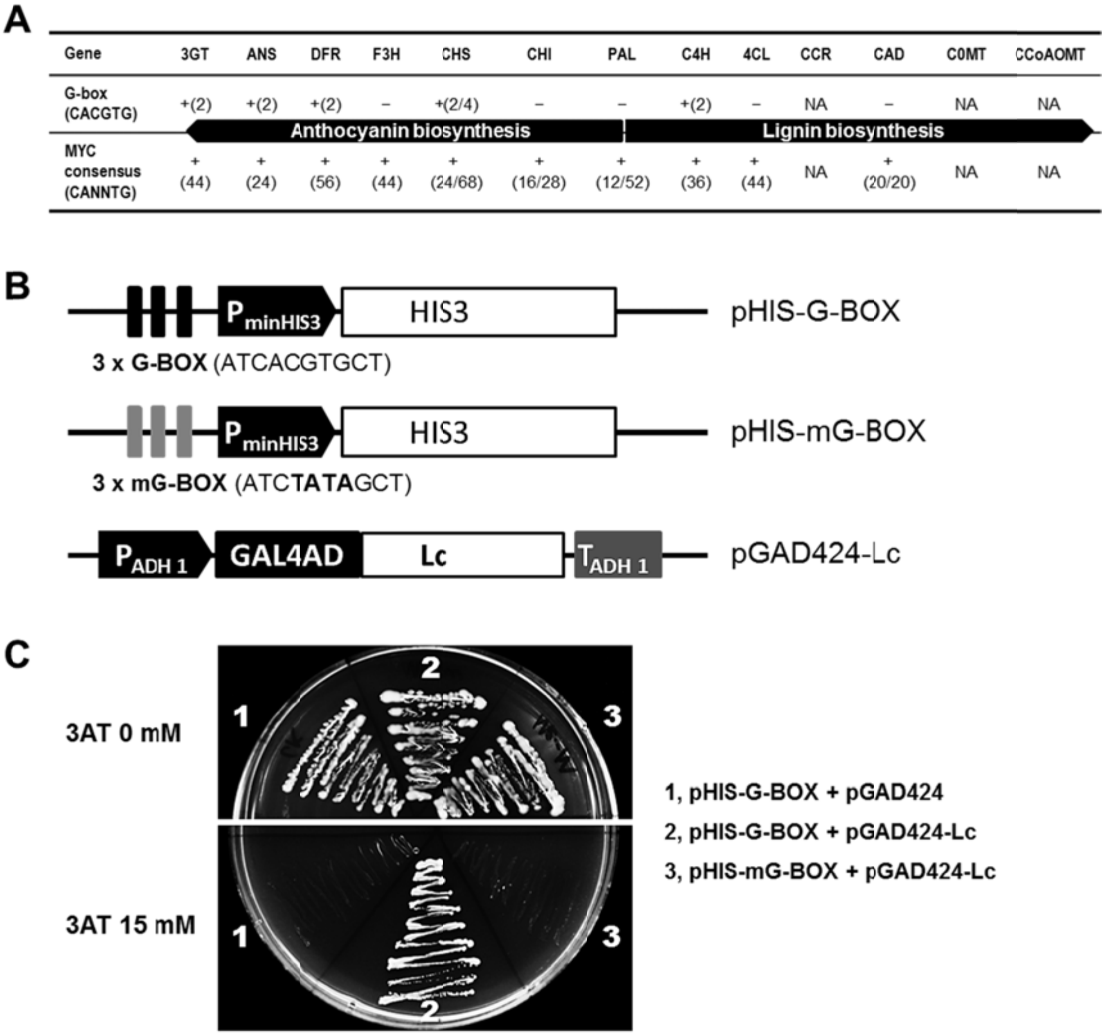

Gene	3GT	ANS	DFR	F3H	CHS	CHI	PAL	C4H	4CL	CCR	CAD	C0MT	CCoAOMT
G-box (CACGTG)	+(2)	+(2)	+(2)	–	+(2/4)	–	–	+(2)	–	NA	–	NA	NA
MYC consensus (CANNTG)	+ (44)	+ (24)	+ (56)	+ (44)	+ (24/68)	+ (16/28)	+ (12/52)	+ (36)	+ (44)	NA	+ (20/20)	NA	NA

Supplementary Figure S3 | The Lc-binding element G-box and MYC consensus in the promoter regions of sweet potato anthocyanin and lignin biosynthetic genes and G-box binding activity by the yeast one hybrid assay. (A) Promoters containing G-box (5′-CACGTG-3′) and MYC consensus (CANNTG) are indicated by "+"; their numbers are indicated within parenthesis and different available homologous gene separated by "/". NA, not available. (B) The bait vector pHIS-G-box and pHIS-mG-box harbor three copies of the regular G-box and mutant (5′-ATC**TATA**GCT-3′) sequences, respectively; the prey vector pGAD424-G-BOX-Lc contains the *Lc* sequence. pGAD424 was used as the negative control. (C) The growth status of the transformed yeast report strain with prey and bait vectors. Only the strain transformed with pHIS-G-box and pGAD424-G-BOX-Lc showed resistance on the SD/-His-Leu plate supplemented with 30 mM 3-amino-1,2,4-triazole (3-AT).

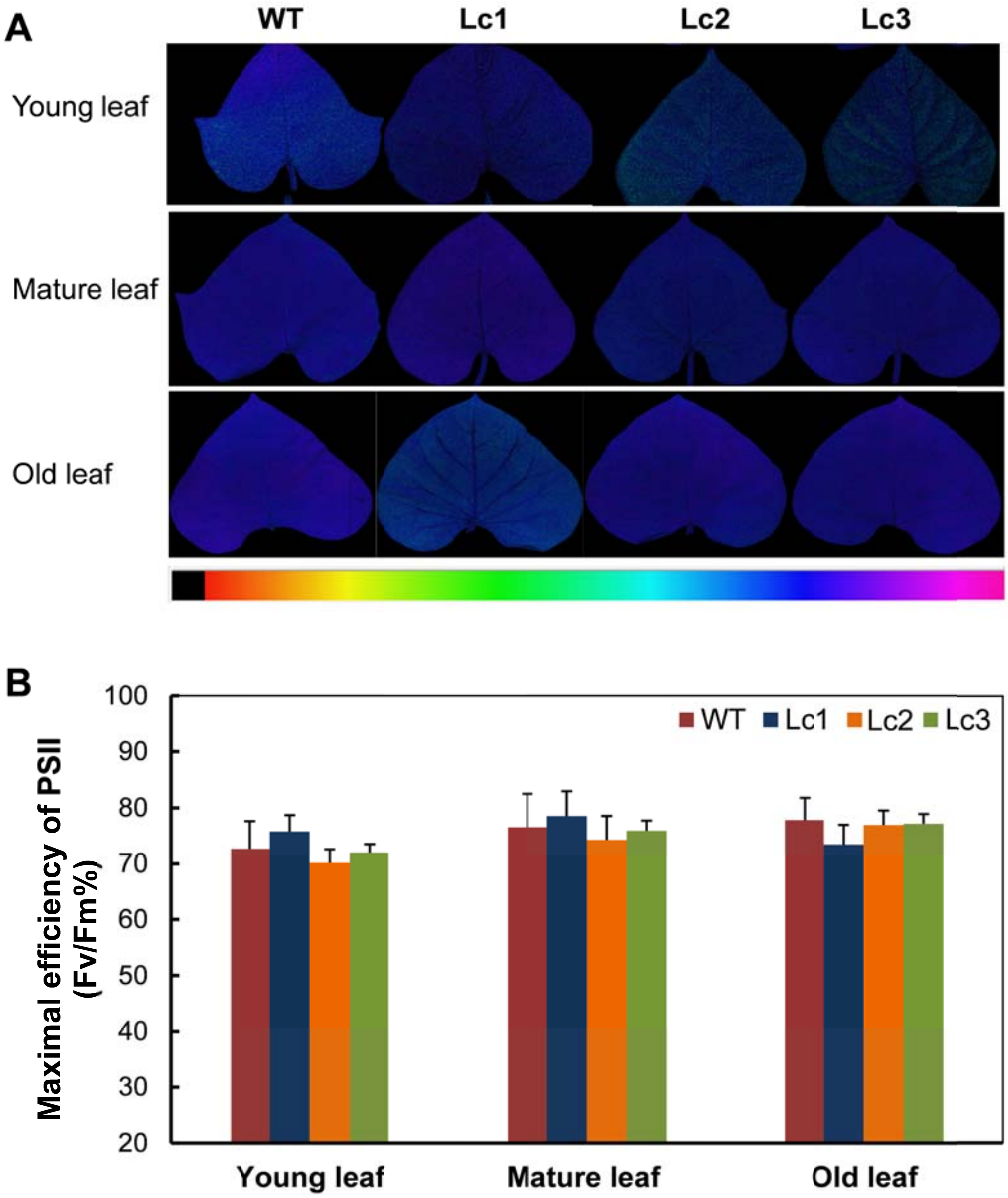

Supplementary Figure S4 | Photosynthesis capacity of young, mature and old leaves in wild-type and *Lc* transgenic sweet potato. (A) Chlorophyll fluorescence. (B) Maximal quantum yield of PSII (Fv/Fm). Error bars represent the SE of three replicates.

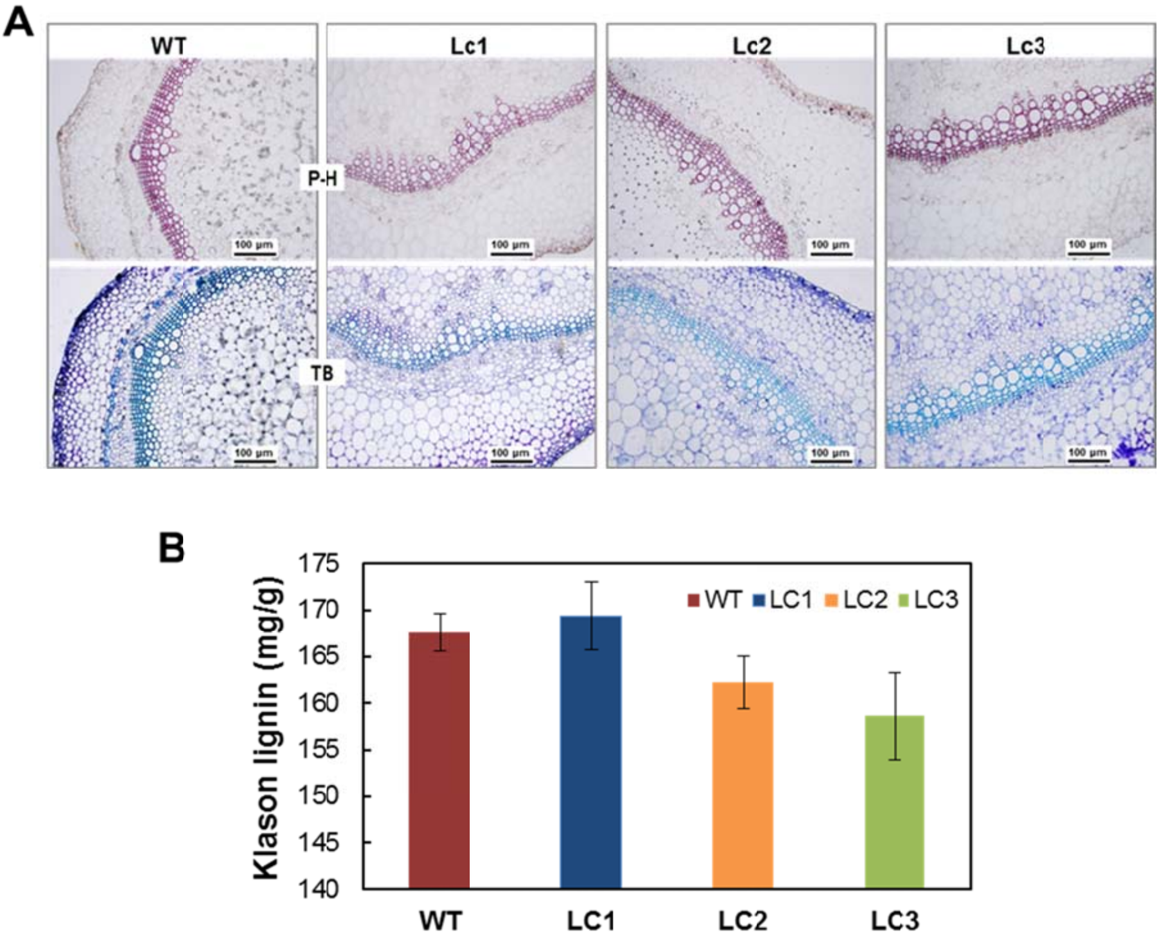

Supplementary Figure S5 | Lignin deposition patterns (A) and Klason lignin content (B) in wild-type and *Lc* transgenic sweet potato. Stem sections of 1.5-month-old plants were stained with two dyes, phloroglucinol-HCl (P-H) and toluidine blue (TB). WT, wild type; Lc1–3, independent *Lc* transgenic lines. Error bars represent the SE of three independent replicates.

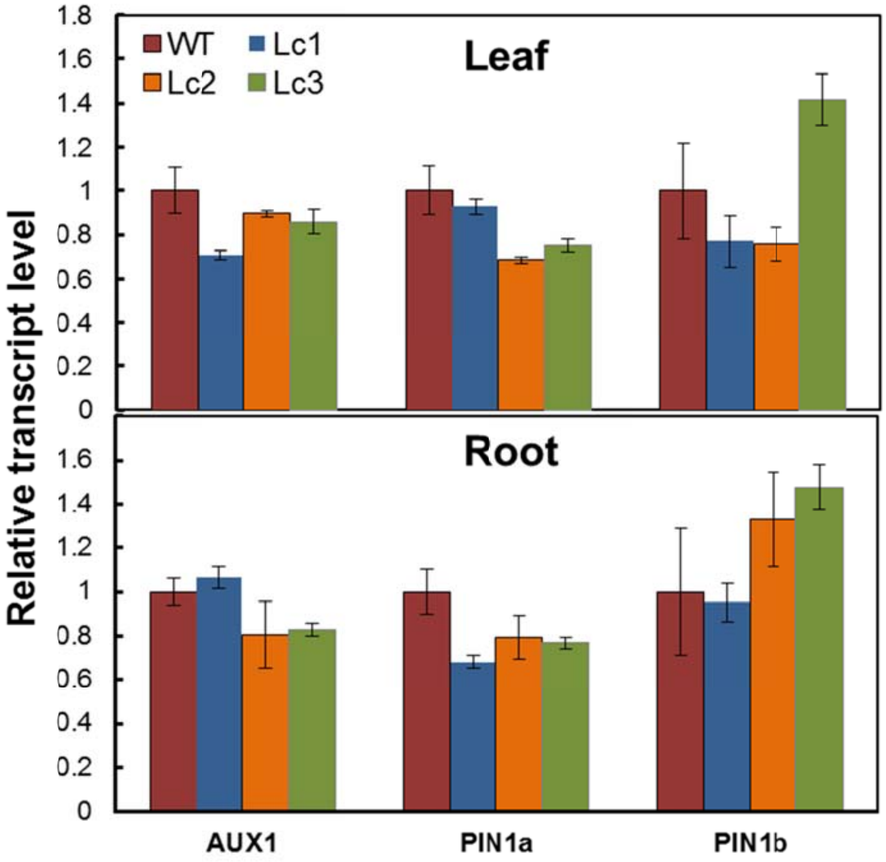

Supplementary Figure S6 | qRT-PCR analysis of the changes in the transcript levels of genes related to auxin transport in leaves and developing storage roots (S16) of 2-month-old wild-type (WT) and *Lc* transgenic sweet potato. Lc1–3, independent Lc transgenic lines. AUX1, auxin transporter protein 1; PIN1a, PIN-FORMED1a; PIN1b, PIN-FORMED1b. Error bars represent the SE of three independent replicates.
